# Growth of Thalamocortical Fibers to the Somatosensory Cortex in the Human Fetal Brain

**DOI:** 10.3389/fnins.2017.00233

**Published:** 2017-04-27

**Authors:** Željka Krsnik, Visnja Majić, Lana Vasung, Hao Huang, Ivica Kostović

**Affiliations:** ^1^Department of Neuroscience, Croatian Institute for Brain Research, School of Medicine, University of ZagrebZagreb, Croatia; ^2^Harvard Medical School, Boston Children's HospitalBoston, MA, USA; ^3^Laboratory of Neural MRI and Brain Connectivity, School of Medicine and Children's Hospital of Philadelphia, University of Pennsylvania PerelmanPhiladelphia, PA, USA

**Keywords:** thalamus, subplate, fiber growth, cortical development, preterm infants

## Abstract

Thalamocortical (TH-C) fiber growth begins during the embryonic period and is completed by the third trimester of gestation in humans. Here we determined the timing and trajectories of somatosensory TH-C fibers in the developing human brain. We analyzed the periods of TH-C fiber outgrowth, path-finding, “waiting” in the subplate (SP), target selection, and ingrowth in the cortical plate (CP) using histological sections from post-mortem fetal brain [from 7 to 34 postconceptional weeks (PCW)] that were processed with acetylcholinesterase (AChE) histochemistry and immunohistochemical methods. Images were compared with post mortem diffusion tensor imaging (DTI)-based fiber tractography (code No NO1-HD-4-3368). The results showed TH-C axon outgrowth occurs as early as 7.5 PCW in the ventrolateral part of the thalamic anlage. Between 8 and 9.5 PCW, TH-C axons form massive bundles that traverse the diencephalic-telencephalic boundary. From 9.5 to 11 PCW, thalamocortical axons pass the periventricular area at the pallial-subpallial boundary and enter intermediate zone in radiating fashion. Between 12 and 14 PCW, the TH-C axons, aligned along the fibers from the basal forebrain, continue to grow for a short distance within the deep intermediate zone and enter the deep CP, parallel with SP expansion. Between 14 and 18 PCW, the TH-C interdigitate with callosal fibers, running shortly in the sagittal stratum and spreading through the deep SP (“waiting” phase). From 19 to 22 PCW, TH-C axons accumulate in the superficial SP below the somatosensory cortical area; this occurs 2 weeks earlier than in the frontal and occipital cortices. Between 23 and 24 PCW, AChE-reactive TH-C axons penetrate the CP concomitantly with its initial lamination. Between 25 and 34 PCW, AChE reactivity of the CP exhibits an uneven pattern suggestive of vertical banding, showing a basic 6-layer pattern. In conclusion, human thalamocortical axons show prolonged growth (4 months), and somatosensory fibers precede the ingrowth of fibers destined for frontal and occipital areas. The major features of growing TH-C somatosensory fiber trajectories are fan-like radiation, short runs in the sagittal strata, and interdigitation with the callosal system. These results support our hypothesis that TH-C axons are early factors in SP and CP morphogenesis and synaptogenesis and may regulate cortical somatosensory system maturation.

## Introduction

Thalamocortical (TH-C) connections are the major source of subcortical input to the cerebral cortex (Jones et al., 1994), and their development is a major focus of current experimental neurobiological (Ghosh and Shatz, 1993; Molnár et al., 1998a,b, 2012; Del Río et al., 2000; Skaliora et al., 2000; Sestan et al., 2001; O'Leary et al., 2007; Little et al., 2009; Chen et al., 2012; Jabaudon and López Bendito, 2012; Price et al., 2012; Garel and López-Bendito, 2014) and imaging studies of the human brain (Huang et al., 2006, 2009; Rados et al., 2006; Aeby et al., 2009; Metzger et al., 2010; Ball et al., 2012, 2013, 2015; Price et al., 2012; Alcauter et al., 2014; Kostovic et al., 2014; Nevalainen et al., 2014; Wang et al., 2015).

Experimental data show that the processes of initial outgrowth, pathfinding, and target selection of TH-C fibers involve complex cellular and molecular interactions, which include a variety of axonal guidance and signaling molecules regulated by specific sets of genes (Sestan et al., 2001; Polleux et al., 2007; Jabaudon and López Bendito, 2012; Molnár et al., 2012; Price et al., 2012; Frangeul et al., 2016). Even though there is evidence that thalamic projections to the cortex play roles in cortical areal differentiation (Sur et al., 1990; O'Leary et al., 1994, 2007), the basic aspects of this thalamic morphogenetic role are not well-understood. The initial basic specification of cortical areas seems to be determined by intrinsic cortical factors, without direct thalamic influence (Rakic, 1988).

In light of the interaction between the thalamus and developing cortex, it is critical that TH-C fibers are engaged in synaptogenesis in the transient cortical compartment—subplate (SP) zone (Molliver et al., 1973; Ghosh et al., 1990; Kostovic and Rakic, 1990; Shatz, 1992; Ghosh and Shatz, 1993; Goodman and Shatz, 1993; Catalano and Shatz, 1998; Hanganu et al., 2001; Kanold, 2004; Zhao et al., 2009; Kanold and Luhmann, 2010). Diverse molecular markers have been identified in this transient synapse-rich compartment (Molnár and Clowry, 2012) during TH-C axon ingrowth. The large transient SP compartment is also known as a “waiting” compartment for TH-C fibers (Rakic, 1977; Kostovic and Rakic, 1984, 1990; Bystron et al., 2008; Kostovic and Judas, 2010) and plays an important role in cortical circuit formation.

Experimental studies have attempted to answer important questions: when and how is sensory input from the thalamus conveyed to sensory cortices and how does sensory thalamic input influence cortical circuitry differentiation (Shatz, 1992; O'Leary et al., 1994; Khazipov and Luhmann, 2006). These questions are of particular interest for human developmental neurobiology where preterm infants are precociously exposed to environmental sensory stimuli, which may influence cortical functions via TH-C connections (Anand and Hickey, 1987; Fitzgerald, 1991, 2005; Lee et al., 2005; Slater et al., 2006; Norman et al., 2008; Hartley and Slater, 2014; Nevalainen et al., 2014, 2015).

The involvement of TH-C system in sensory-driven cortical activity at an early preterm age (24–26 PCW) is not surprising since TH-C fibers are already well-developed during the human midfetal period (Marin-Padilla, 1970; Kostovic and Goldman Rakic, 1983; Kostovic and Rakic, 1984; Mojsilovic and Zecevic, 1991; Hevner, 2000; Kostovic and Judas, 2002, 2010; Kostovic and Jovanov-Milosevic, 2006), when they establish synaptic contacts in the transient SP zone (Kostovic and Rakic, 1990; Kostovic and Judas, 2006; Kanold and Luhmann, 2010; Hoerder-Suabedissen and Molnár, 2015). In addition, TH-C fiber penetration of the CP and subsequent synaptogenesis in the human brain occurs as early as 23 PCW (Molliver et al., 1973; Kostovic and Molliver, 1974; Kostovic and Judas, 2007). Growing anatomical evidence of TH-C connections in the human preterm brain (Kostovic and Judas, 2010) provides a solid basis for the interpretation of recent magnetic resonance (MR) studies showing early development of the TH-C connectoma (Berman et al., 2005; Rados et al., 2006; Aeby et al., 2009; Metzger et al., 2010; Ball et al., 2012, 2013, 2015; Price et al., 2012; Alcauter et al., 2014; Kostovic et al., 2014; Nevalainen et al., 2014; Gao et al., 2015; Wang et al., 2015), as well as early involvement in the default network and somatosensory integration (Fransson et al., 2009, 2011; van den Heuvel and Hulshoff Pol, 2010; Hoff et al., 2013; van den Heuvel et al., 2015).

Numerous functional studies regarding early TH-C relationships in preterm infants analyzed the somatosensory component of the TH-C system (Nevalainen et al., 2014, 2015), but anatomical data on this part of the developing TH-C connections are limited and preliminary (Kostovic et al., 1980). The precise timing and choreography of growing TH-C fibers to the prospective somatosensory cortex are not known. Investigation of the topographical relationships of TH-C somatosensory fibers within the different segments of the white matter (internal capsule, crossroads, sagittal strata, centrum semiovale, and corona radiata; for terminology see Kostovic et al., 2014) is also needed for future studies of the selective, topographically defined vulnerability of developing white matter. Normative data on the timing, growth pattern, and ingrowth in the cortex is important for studying abnormal cortical development. Here we try to answer some of these questions using AChE histochemistry, which reliably and selectively stains several classes of TH-C fibers arising from sensory and some associative nuclei (Kostovic and Goldman Rakic, 1983; Kostovic and Rakic, 1984) in fetal material ranging from the end of the embryonic period at 7.5 PCW to the establishment of adult-like relationships at 34 PCW (Zagreb Neuroembryological Collection, www.zagrebbraincollection.hr).

Findings from the complete developmental series of the human brain will provide information about the early origin, timing of growth, trajectory along crucial topographical points, relationships with other fiber systems, and involvement in lamination of the SP and CP of the somatosensory cortex. These normative data will be essential for interpreting developmental intrauterine and perinatal lesions of the TH-C connectoma, as well as the more subtle abnormalities that can lead to developmental disorders such as autism and schizophrenia.

## Materials and methods

Different TH-C axons growth phases (outgrowth, initial path-finding, crossing, spread and “waiting” in the SP, cortical target selection via accumulation in SP, and ingrowth in the CP were analyzed using histological sections from fixed, post-mortem embryonic, fetal, and preterm brains ranging from 7 to 34 postconceptional weeks (PCW). Examined brains are part of Zagreb Neuroembryological Collection and University of Maryland Brain and Tissue Bank. Brain specimens were obtained from medically indicated or spontaneous abortions at several clinical and pathological departments of the University of Zagreb, School of Medicine, Zagreb, Croatia. Informed consent was provided, and procedures were approved by the corresponding Institutional Review Boards. Fetal age was estimated on the basis of crown-rump length (CRL, in mm) and pregnancy records.

Brains were fixed by immersion in 4% paraformaldehyde in 0.1 M phosphate-buffered saline (PBS, pH 7.4) and tissue blocks were either frozen or embedded in paraffin wax. Sections were processed with histological Cresyl violet (Nissl) staining (to delineate cytoarchitectonic boundaries) or histochemical (AChE) or immunohistochemical (anti-fibronectin) methods (Kostovic et al., 2014).

For AChE histochemistry, sections were incubated according to Lewis's modification of the Koelle-Friedenwald acetylthiocholine iodide method. The reaction product was developed with sodium sulfide in 0.2 M acetic acid after incubation for up to 48 h (Kostovic and Goldman Rakic, 1983). The AChE histochemistry method was used to visualize a subset of growing thalamocortical afferents and certain sagittally oriented axon strata, including the external capsule.

For immunohistochemical staining, following deparaffinization and pretreatments with 0.3% hydrogen peroxide and blocking solution, sections were incubated with the primary antibody anti-fibronectin (1:400; F3648, Sigma-Aldrich, St. Louis, MO, USA). Secondary biotinylated anti-rabbit antibody from Vectastain ABC kit (Vector Laboratories, Burlingame, CA, USA) was used according to the manufacturer protocol, and visualization of peroxidase activity was done using 3,3-diaminobenzidine with metal enhancer (Sigma, St. Louis, MO, USA). Stained sections were coverslipped with Histamount (National Diagnostics, Charlotte, NC, USA). Negative controls were performed by replacing the primary antibody solution with blocking solution during the incubation procedure. For detailed methodology see Kostovic et al. (2014).

Sections were scanned by the high-resolution digital slide scanner NanoZoomer 2.0RS (Hamamatsu, Japan) and obtained images were compared with images of postmortem diffusion tensor imaging (DTI) tractography (code No NO1-HD-4-3368 and NO1-HD-4-3383) from Maryland Brain and Tissue Bank. Figures were assembled in Microsoft Publisher (Microsoft, Redmond, WA, USA).

Histology images were compared to the six postmortem diffusion MR images from the Maryland Brain and Tissue Bank (code No NO1-HD-4-3368 and NO1-HD-4-3383). Postmortem brains were fixed with 4% paraformaldehyde. Forty-eight hours before scanning, the fixative was washed out with PBS. 3D multiple spin echo DTI and T1-weigthed MRI images were obtained using 11.7-T (specimens <16 PCW) or 4.7-T Bruker scanners (specimens >16 PCW). Diffusion-weighted images (DWIs) were acquired in seven linearly independent directions. For the acquisition of diffusion tensor images with 11.7 MR, we used the following parameters: *b* = 1,000 s/mm^2^, TE (time to echo) = 67 ms, TR (repetition time) = 0.8 s, FOV (field of view) = (25–35 mm) × (25–35 mm) × (25–35 mm), imaging matrix = 128 × 80 × 80, with an imaging resolution = 200–400 μm. Imaging parameters of older specimens, using a 4.7-T scanner, were as follows: *b* = 1,000 s/mm^2^, *TE* = 66 ms, *TR* = 0.8 s, *FOV* = (40–52 mm) × (40–52 mm) × (40–52 mm), and imaging matrix = 128 × 72 × 72, with an imaging resolution of 300–600 μm.

White matter tracts were reconstructed in 3D using a previously described continuous tracking method (Mori et al., 1999). For fiber tract reconstruction, we used MRIStudio (https://www.mristudio.org). To reconstruct thalamocortical and callosal fiber tracts, we used a single region of interest (ROI) with a fractional anisotropy threshold of 0.15. The seed regions (ROIs) were manually delineated using the anatomical landmarks previously described on MR images (Kostovic and Vasung, 2009). The entire thalamus of one hemisphere was used as an ROI for thalamocortical tract reconstruction. The corpus callosum ROI was manually delineated in the mid-sagittal plane and adjacent parasagittal slices (*n* = 3).

*In vivo* imaging was performed by fast T2-weighted (HASTE) MR imaging on a 1.5-T device (Magnetom Symphony; Siemens, Erlangen, Germany) and was provided by Prof. Marko Rados.

## Results

**Figure 1:**
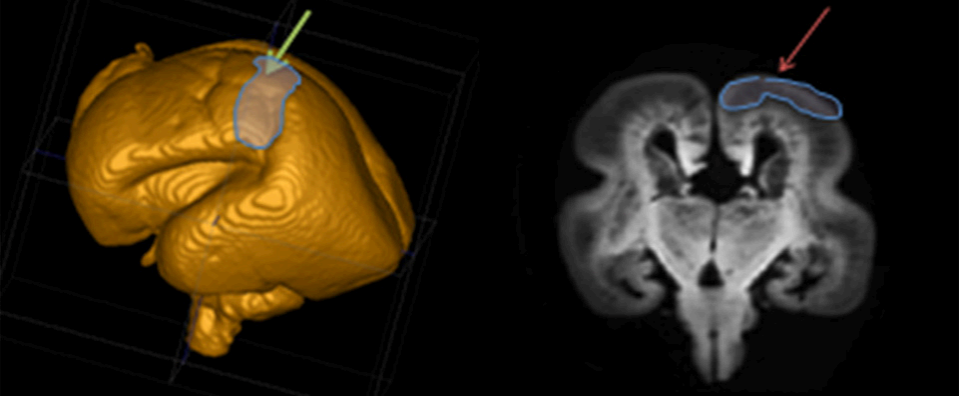
**Position of the prospective somatosensory cortex based on 3D MRI reconstruction of the post mortem specimen**.

The TH-C fibers in human brain show a remarkably prolonged period of growth (from 7 to 34 PCW). To follow all phases of fiber growth and delineate trajectories, we divided the fetal period into 8 phases. The position of the prospective somatosensory cortex in specimens older than 15 PCW was determined based on 3D reconstruction on MRIs of postmortem specimen (Figure 1). In younger specimens, the midlateral telencephalic pallium was approximately considered as the prospective somatosensory cortex.

### Phase 1 (7.5 PCW)

**Figure 2:**
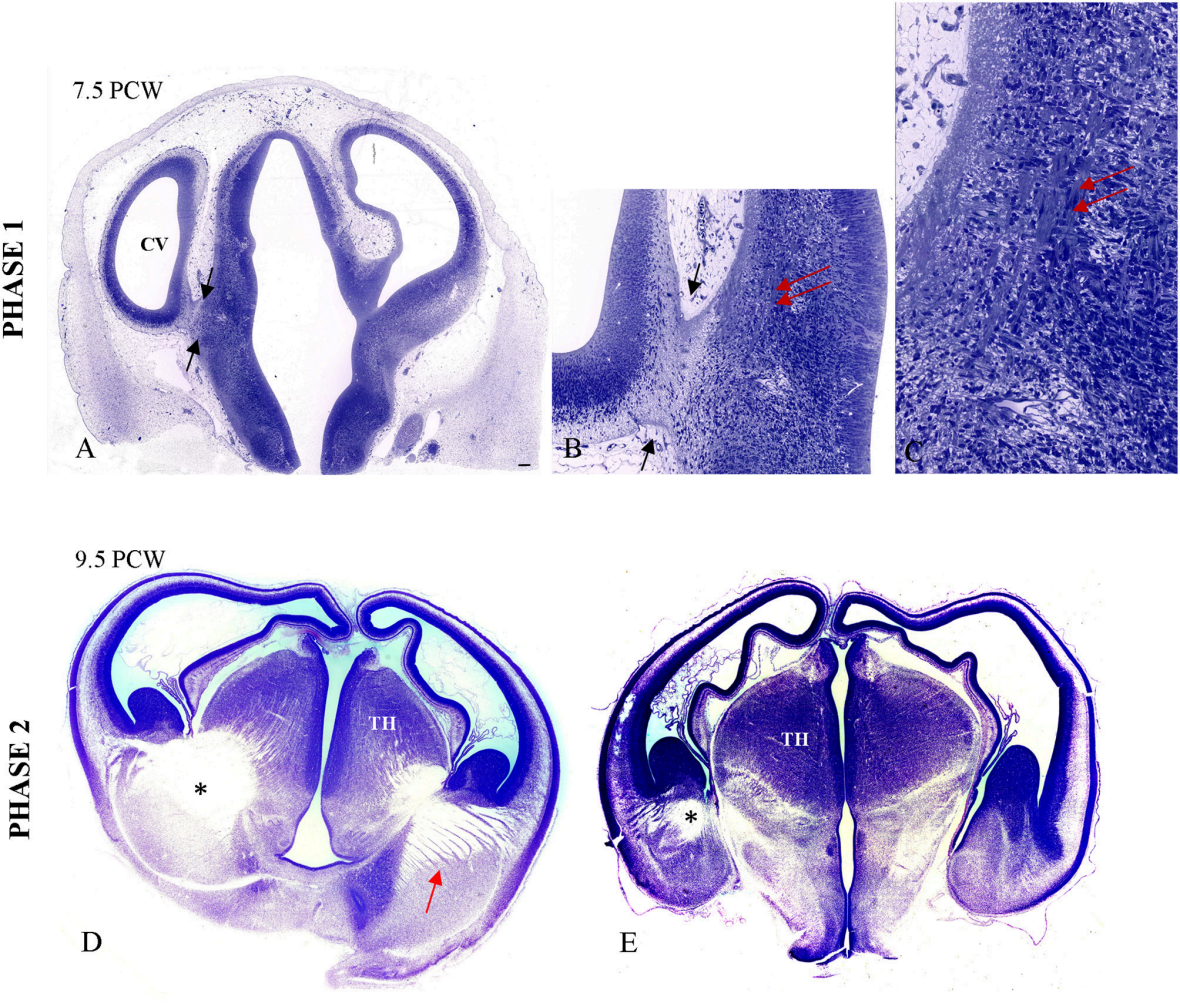
**Primitive TH-C stalk at 7.5 PCW (A,B; between arrows)**. The bundles of the thalamic axons in the ventrolateral aspect of thalamus are visible on high magnification (**B,C;** double arrow), on 1 μm-thick plastic section. The fiber bundles are stained due to osmification of the tissue prepared for electron microscopy. **(D,E)** The massive TH-C bundle (^*^) stretching from the ventrolateroposterior aspect of the thalamus, below the diencephalo-telencephalic sulcus and “arriving” below the ganglionic eminence at the pallio-subpallial boundary in the radiating fashion at 9.5 PCW on Nissl-stained celloidin sections (arrow). TH, thalamus. Scale bar: 100 μm.

The first phase of TH-C axon growth begins in the form of “pioneering” axonal fascicles as early as 7.5 PCW in the ventrolateral part of the thalamic anlage (Figures 2A,B,C), below the diencephalo-telencephalic sulcus, just across the caudal-basal division of the telencephalic ganglionic eminence. At the topographical transition between the diencephalon and telencephalon, there is formation of the cerebral stalk comprised of fibrillar tissue (described by His, 1904; Hochstetter, 1919) that is circumvallated by the deep diencephalo-telencephalic sulcus. The description of this first phase of TH-C fiber growth is based on studies of serially sectioned 1-μm-thick plastic sections (specimen CF 120 from Zagreb Neuroembryological Collection), which is CRL 20 mm, or 7.2 weeks according to Olivier/Pinot ovulation age and roughly corresponding to horizon 20 (18–22 mm CRL) of Streeter.

### Phase 2 (8–9.5 PCW)

During the second phase, axons from the ventrolateral thalamus form massive bundles during initial path-finding and crossing of the diencephalic-telencephalic boundary (specimen CF 96, Zagreb Neuroembryological Collection). This bundle shows initial radiation toward the telencephalon, forming a trapezoid fan-like structure with thick bundles of axons of different length whereas some bundles run just below the ganglionic eminence, reaching the pallial-subpallial boundary. Figures 2D,E show massive TH-C bundles stretching from the ventrolateroposterior aspect of the thalamus below the diencephalo-telencephalic sulcus and “arriving” below the ganglionic eminence at the pallio-subpallial boundary in a radiating fashion.

### Phase 3 (9.5–11 PCW)

**Figure 3:**
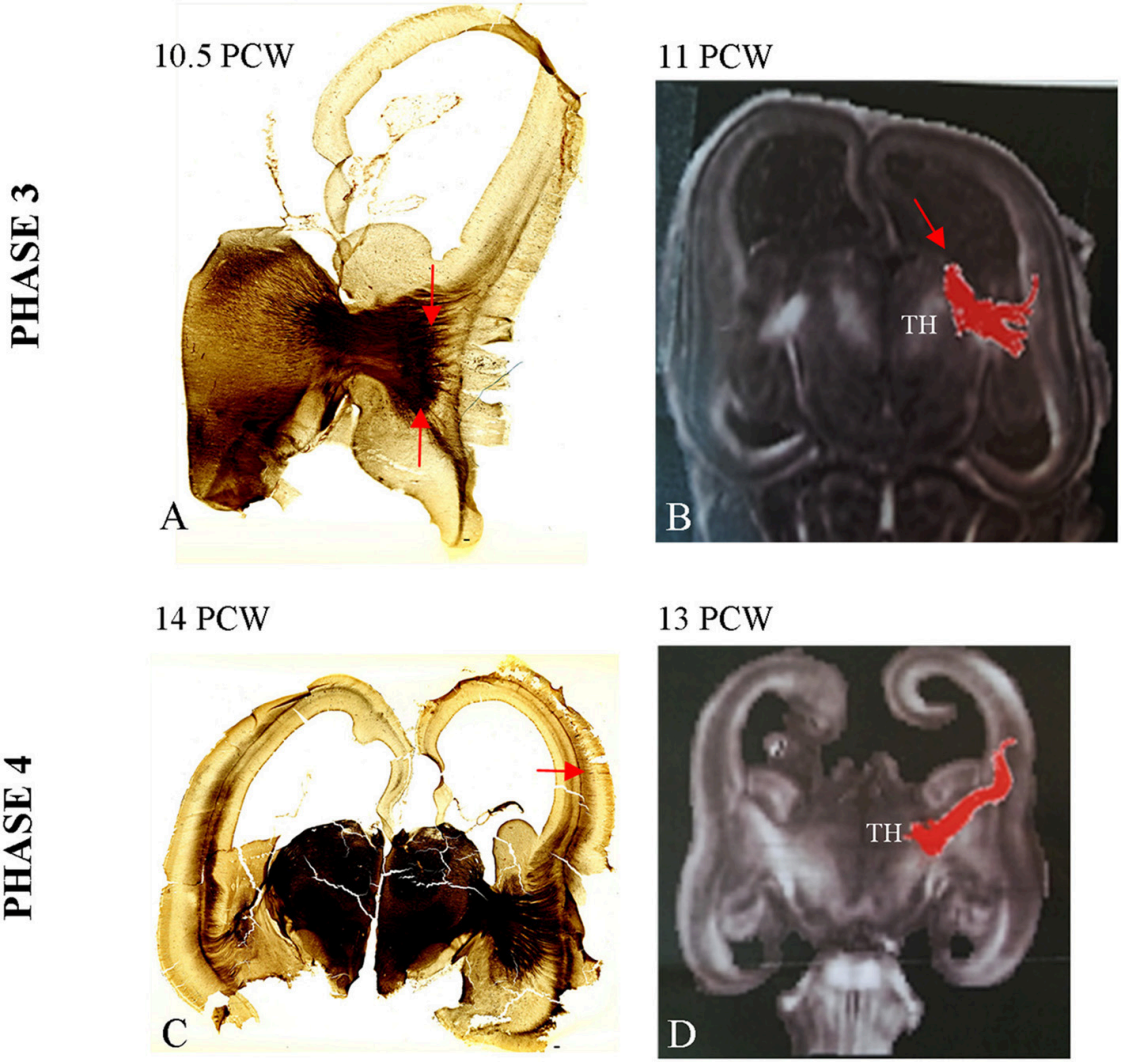
**Strongly stained radiating TH-C bundle (between arrows, A)** approaching the pallium beyond the pallial-subpallial boundary. The DTI image of 11 PCW **(B)** shows DTI reconstruction of the TH-C bundle corresponding to the histological section from **(A)**. **(C)** The AChE reactive fibers originated from thalamus and basal forebrain enter the deepest portion of loose deep CP at 14 PCW (arrow). See also data from Duque et al. (2016). **(D)** Shows DTI image of extension of TH-C fibers directed to deep portion of CP at 13 PCW. Scale bar: 100 μm.

During the third phase, TH-C axons pass the crucial periventricular crossing area at the pallial-subpallial boundary and enter the intermediate zone in a radiating fashion, from ventral and medial to dorsal and lateral (Figure 3A). This thick TH-C fiber system can also be readily demonstrated by DTI (Figure 3B). TH-C fiber systems are aligned with fibers from the basal forebrain after crossing the pallial-subpallial boundary. AChE staining more precisely demonstrates the TH-C fibers at their radiation toward the prospective somatosensory cortex. Thus, AChE preparation allows fibers to be followed from the moderately AChE-reactive ventral posterolateral (VPL) territory toward the midlateral cortex where TH-C fibers show radiation on both coronal and horizontal sections. The fibers are grouped in upper and lower sectors, forming a V-shape. The main body of the AChE-stained TH-C radiation on the coronal sections appears trapezoidal. The fiber bundles within the TH-C radiation are thinner than those observed at earlier stages. The immature internal capsule on the horizontal sections also shows a V-shaped form, open laterally with anterior (rostral) and posterior (caudal) limbs. On both coronal and horizontal planes, thalamic fibers encompass the developing putamen of the corpus striatum. In this phase, radial growth of TH-C fibers destined for the somatosensory cortex is rather direct, and fibers run in the sagittal stratum for a short distance. This is in contrast to TH-C fibers for frontal and occipital cortical regions that run within sagittal strata of the intermediate zone for longer distances. The radiating trajectories of growing TH-C fibers actually obscure delineation of the intermediate zone, a distinct lamina of cerebral wall at this midlateral level. Thus, the intermediate zone shows discontinuity between the occipito-parietal and frontal portions of the cerebral wall. Analysis of the AChE-stained coronal and horizontal sections reveals that there are already widely open V-shaped fibers lateral to the thalamus, resembling a primitive internal capsule (Figure 3A).

### Phase 4 (12–14 PCW)

During the fourth phase there is an increase in AChE reactivity of the VPL thalamus and further elongation of TH-C axons that grow for a short distance within the deep sagittal stratum of the intermediate zone. The AChE-reactive fibers originating from the basal forebrain form the more superficial stratum within the external capsule (Figure 3C). Some TH-C axons enter the deepest, loose portion of the CP and participate in the formation of so-called “second plate” (Kostovic and Rakic, 1990). The process of early TH-C fibers ingrowth to the deepest portion of the CP (second) is closely related to subsequent SP expansion (Duque et al., 2016). DTI images show that TH-C fibers have already reached the deep CP (Figure 3D). This is actually the first, transient sublamination of the deep CP (Duque et al., 2016), after which the CP undergoes secondary condensation (Kostovic and Rakic, 1990). Due to deepening of the lateral cerebral wall in the location of the future Sylvian fossa, the TH-C fiber system appears more bifurcated than in the previous phase. Moreover, the upper, more dorsal bundles approach the somatosensory cortex, while lower (ventral) bundles approach the prospective temporal cortex and participate in the initial corona radiata.

### Phase 5 (14–18 PCW)

**Figure 4:**
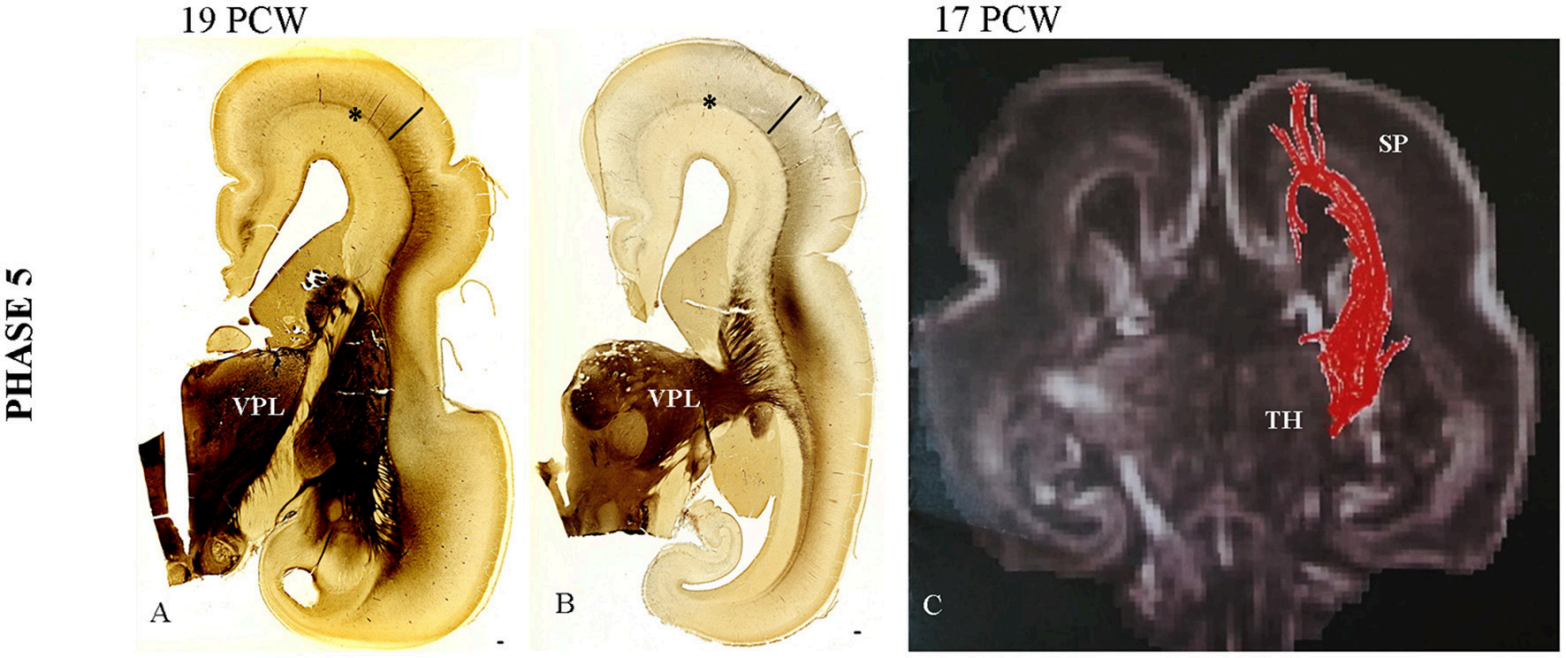
**Gradual spread of the AChE-reactivity within the deep subplate at 19 PCW (A). (B)** The same process at more caudal level. **(C)** The DTI extension of the TH-C fibers through SP at 17 PCW. Asterisk marks external capsule; bar marks SP. Scale bar: 100 μm.

**Figure 5:**
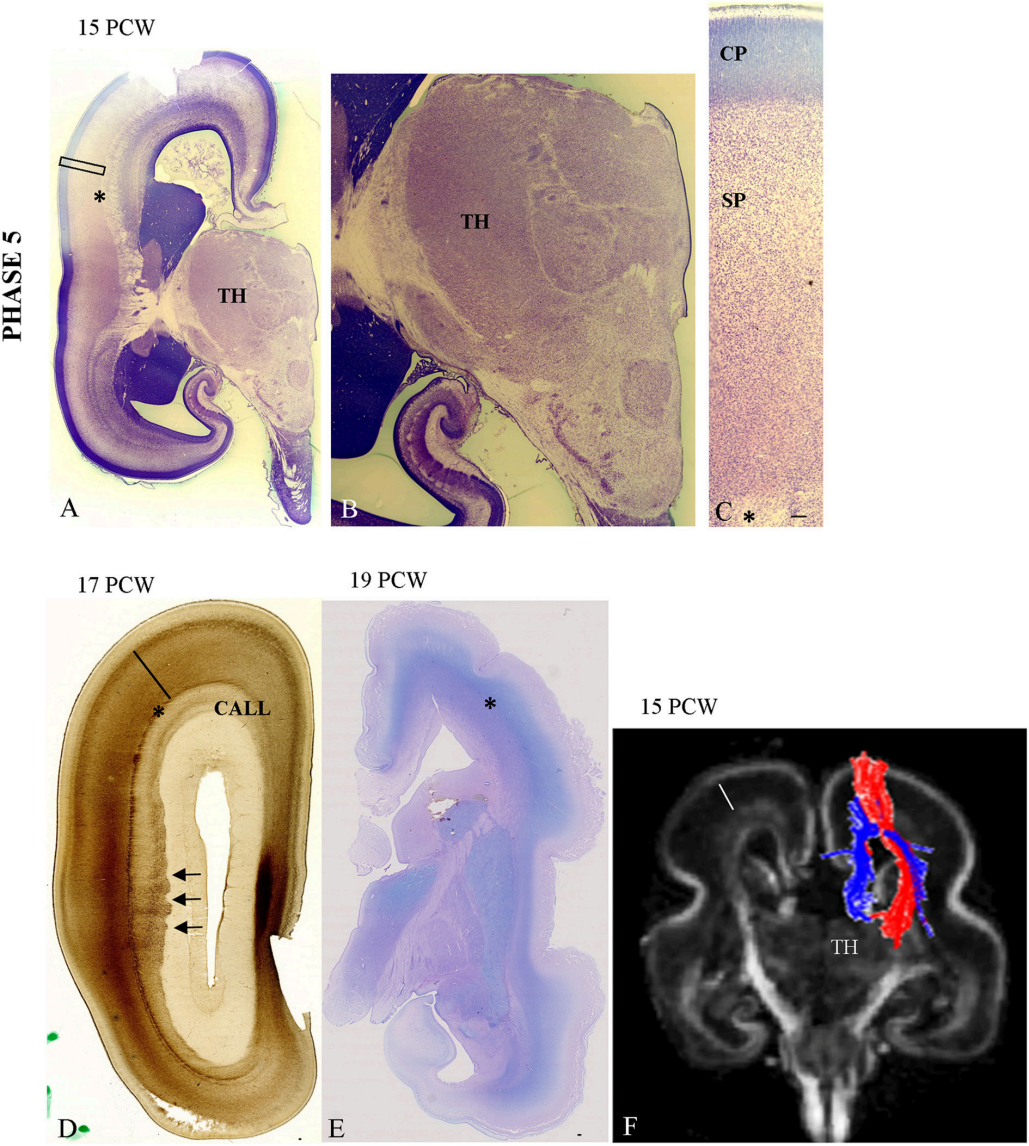
**The cytoarchitectonic differentiation of the VPL nuclei at 15 PCW (A,B)** and further cytoarchitectonic differentiation of CP and SP (**C**—Nissl stained section). The vertically aligned embryonic “columns” and cell-sparse SP with cells showing advanced cytological maturation **(C)**. Close interdigitation appears between callosal (AChE negative) and thalamic (AChE reactive) fibers (**D**, arrows). **(E)** Periodic acid-Schiff (PAS)-Alcian staining at 19 PCW (compartment between asterisk and poorly-stained CP is SP). Asterisk marks border between SP and intermediate zone. **(F)** Shows DTI reconstruction of interdigitation of TH-C (red) and callosal fibers (blue) at 15 PCW. Scale bar: 100 μm.

During the fifth phase there are two important histogenetic events:

1. The TH-C fibers, after short run in the sagittal stratum, spread gradually throughout the deep portion of the SP, approaching their cortical target (Figures 4A–C).
2. Close interdigitation appears between callosal (AChE-negative) and thalamic (AChE-reactive) fibers (Figure 5D).

**Figure 6:**
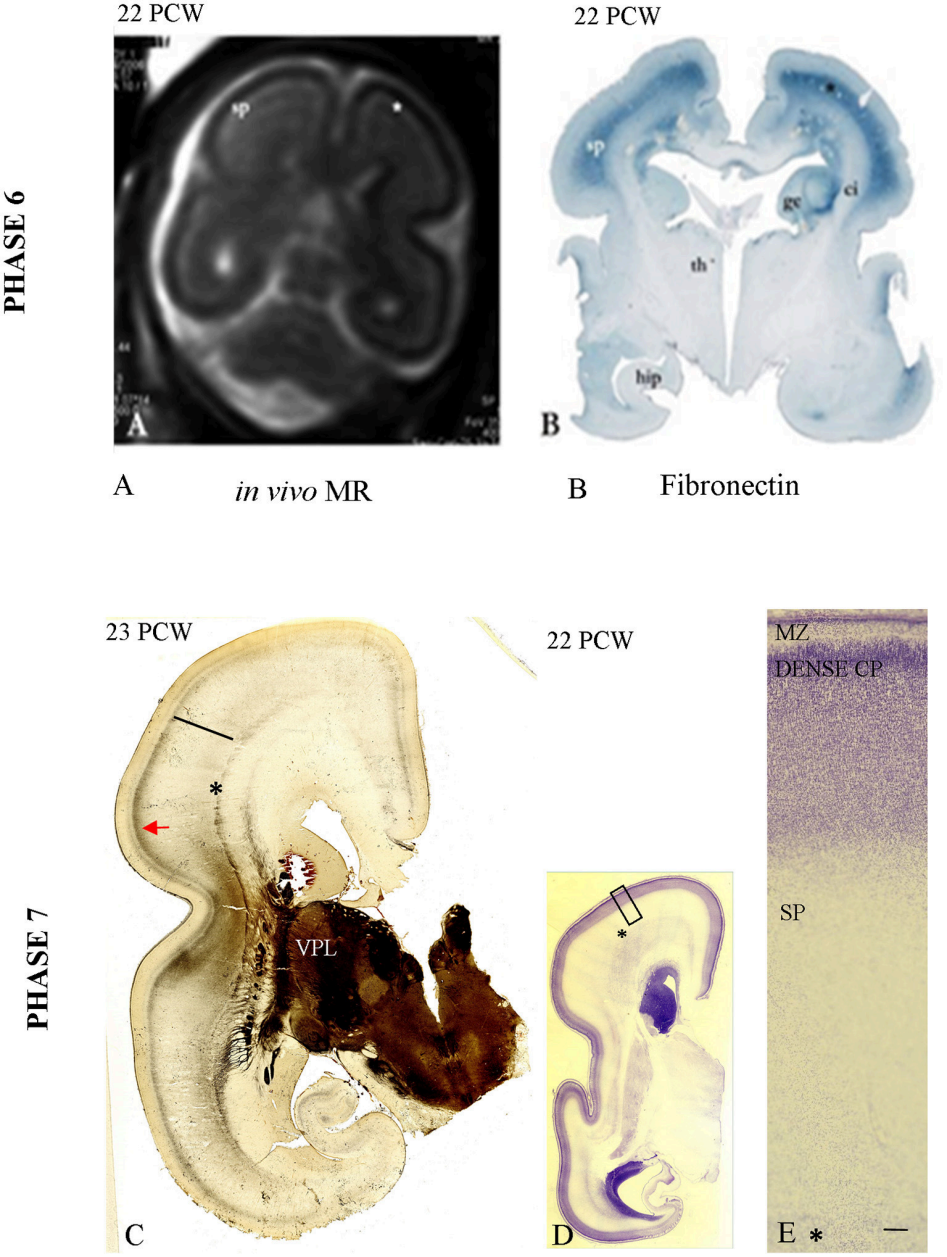
***In vivo*** **image of the fetal telencephalon at 22 PCW (A);** accumulation of TH-C fibers in superficial SP marked with (^*^) corresponding to the fibronectin-immunoreactive SP [**B**, already presented by Kostovic et al. (2006)]. **(C)** Shows the first AChE reactive band in the prospective somatosensory CP while the remaining AChE reactive fibers are still in the superficial SP (arrow). (**D,E**: The initial lamination of the CP on Nissl preparation (low and high magnification). Asterisk marks border between SP and fiber strata of the intermediate zone (fetal white matter); bar marks SP. Scale bar: 100 μm.

The first event of gradual expansion of AChE reactivity within the deep SP (Figures 4A,B) starts with relatively weak staining (Figures 4A,B). Later, AChE reactivity occupies the whole extent of the SP zone (Figure 5D), corresponding to the plexiform, synaptic compartment of the SP as previously defined by Kostovic and Rakic (1990). Fibrillar and Nissl cytoarchitectonic borders were used to delineate the SP layer during this phase. The most reliable marker for delineation for deep SP was the external capsule (Figures 4A,B, 5D, asterisk) that is the outermost fiber sublayer; it runs in a sagittal direction and is strongly AChE reactive (Kostovic, 1986; Kostovic et al., 2002). The upper border of the SP is at the bottom of the cell-dense CP. As additional markers, we used conventional Periodic acid-Schiff (PAS)-Alcian staining (Figure 5E) and fibronectin immunoreactivity (Figure 6B). During TH-C fiber invasion and spread within the SP, there is advanced differentiation of the ventrolateral thalamic territory (Figures 5A,B), concomitantly with SP differentiation, while the CP still shows columnar arrangement without clear lamination (Figure 5C). AChE reactivity clearly defined the ventrolateroposterior territory of the thalamus (Figures 4A,B), dorsomedial nucleus (Kostovic and Goldman Rakic, 1983), and pulvinar posterior complex (Kostovic and Rakic, 1984). The second event of interdigitation of TH-C and callosal fibers is most visible during this developmental period. The interdigitation of AChE-positive TH-C fibers and AChE-negative callosal fibers (arrows) are clearly visible on AChE preparations (Figure 5D, arrows). DTI reconstruction of the interdigitation of TH-C (red) and callosal fibers (blue) is shown in Figure 5F.

### Phase 6 (19–22 PCW)

Accumulation of TH-C fibers in the superficial SP compartment, below the somatosensory target cortical area, is the main histogenetic event during this phase. AChE reactivity during this period helps subdivision of the SP into the superficial and deep layers, with the superficial SP exhibiting stronger AChE reactivity. TH-C fiber accumulation is also visible on *in vivo* MR images in the form of higher signal intensity (SP on Figure 6A) and on immunohistochemically stained sections for fibronectin (Figure 6B). *In vivo* imaging was previously presented in the pilot study by Kostovic et al. (2006).

### Phase 7 (23–24 PCW)

During this phase of penetration of the target CP and initial CP lamination (Figures 6D,E), the TH-C AChE-reactive fibers gradually penetrate the prospective somatosensory cortex, forming a new band in the middle of the CP (Figure 6C). This is the first AChE-reactive band in the prospective somatosensory CP. The remaining AChE-reactive fibers are still accumulating in the superficial SP (Figure 6C, arrow).

### Phase 8 (25–34 PCW)

**Figure 7:**
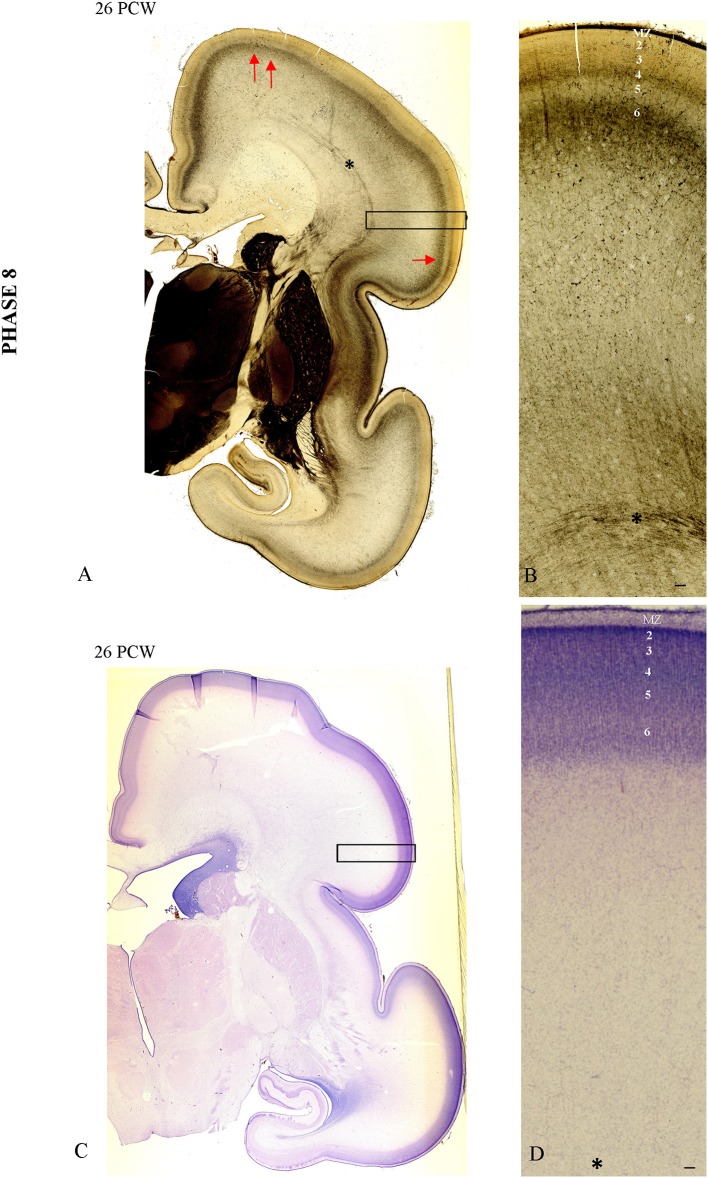
**The AChE patterned lamination in the “ventral” somatosensory cortex (arrow) precedes the AChE lamination in the dorsal cortex (double arrow, A)**. In the “ventral” somatosensory cortex at 26 PCW reactivity is trilaminar (CP AChE-positive band, CP AChE-negative band and superficial SP AChE-positive band), shown on **(B)**. Adjacent Nissl stained section with prospective cortical layers **(C,D)**. Asterisk marks border between SP and fiber strata of intermediate zone (fetal white matter). Scale bar: 100 μm.

During this phase of the elaboration and address selection in the CP, there is an increase in AChE laminar pattern staining (Figure 7A). In the “ventral” somatosensory cortex (arrow), the AChE reactivity is more prominent than the weak AChE lamination pattern in the dorsal cortex (Figure 7A, double arrow). In the somatosensory cortex, reactivity is trilaminar (CP AChE-positive band, CP AChE-negative band, and superficial SP AChE-positive band; Figures 7A,B). The AChE-laminated pattern of the CP and superficial SP is shown on high magnification in Figure 7B. An adjacent Nissl-stained frozen section shows the cytoarchitecture of the CP with the initial 6-layer pattern (Figures 7C,D).

**Figure 8:**
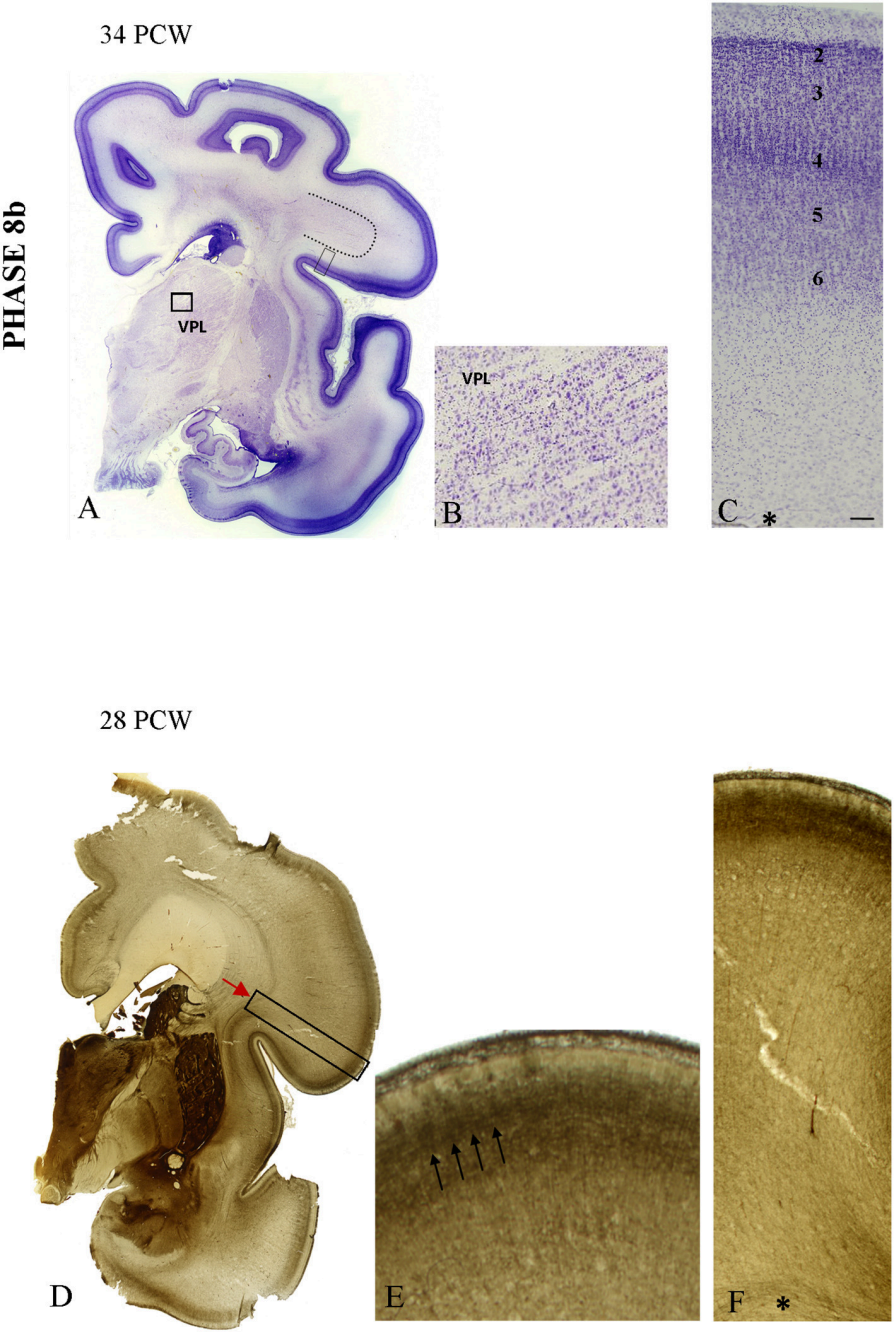
**The appearance of the initial prospective 6-layered pattern on Nissl preparation at 34 PCW (A,C)**. The border between the developing gyral white matter and SP became obscured (dotted line on **A**) due to radial arrangement of fibers. The advanced cytoarchitectonic differentiation of the VPL territory is shown on **(B)**. **(D–F)** The increase in elaboration of AChE activity in the form of the uneven pattern suggestive of the vertical banding at 28 PCW. On **(E)** three AChE reactive strata are seen: superficially in the MZ, in the middle of the CP and the deep interface of SP and CP. The middle band shows uneven staining in both tangential and radial direction, suggestive of vertical patterning (arrows). Asterisk marks border between SP and fiber strata of intermediate zone. Scale bar: 100 μm.

In specimens older than 28 PCW, the 6-layered cortical pattern is visible in the CP on Nissl-stained sections (Figures 8A,B,C). The increase and elaboration of AChE reactivity in the middle of CP exhibits an uneven pattern suggestive of vertical banding (Figures 8D,E). Vertical “bands” are between 200 and 260 μm wide and are separated by narrow (<10 μm) “septa.” AChE reactivity in the middle of the CP is much stronger than in the underlying SP (on the order of 3 × and more). Advanced cytoarchitectonic differentiation of the VPL territory is shown in Figure 8B.

In conclusion, our results are in line with the findings on the growth schedule and tempo of development of TH-C axons to frontal and occipital areas (Kostovic and Goldman Rakic, 1983; Kostovic and Rakic, 1984). These observations confirm their prolonged growth (lasting 4 months), as well as their parallel growth with the basal forebrain afferents. However, ingrowth of fibers to the somatosensory cortex seems to precede ingrowth to the frontal and occipital areas for almost 2 weeks. In addition, the TH-C axons destined for the somatosensory cortex show characteristic radiating trajectories, shorter runs in sagittal strata, and interdigitation with the callosal fiber system before entering the SP and ingrowth to the CP. We propose that early arrival of TH-C and basal forebrain fibers to the deep CP is an important morphogenetic factor for spreading of deep CP and formation of the SP. Similarly, their interaction with the growth of corticocortical (callosal) fibers may serve as a morphogenetic factor for callosal afferent growth. The interaction between TH-C axons and SP neurons during prolonged midfetal growth is crucial for establishing early transient fetal circuitry. Simultaneous interaction of TH-C axons with the SP and CP neurons after 24 PCW explains different transient electrophysiological phenomena such as large electric waves and form an anatomical basis for early nociceptive influence on the cortex.

## Discussion

This paper presents new evidence on the timing, growth, and fiber trajectories of prospective somatosensory TH-C fibers in the developing human fetal brain and the spatial relationship of TH-C fibers within different components of the developing white matter. We confirmed previous observations on laminar distribution in the transient SP zone and developing CP as important indicators of TH-C fiber involvement in morphogenetic and functional interactions in the developing cerebrum. In addition, we show normative histological and MR postmortem data useful for study of developmental vulnerability of the TH-C system.

### Timing and pattern of growth

The early appearance of the massive fiber system observed in the ventrolateral anlage of the human thalamus during the late embryonic and early fetal periods is consistent with the classical description of the developing human thalamus (His, 1904; Hochstetter, 1919; Gilbert, 1935; Cooper, 1950; Dekaban, 1954; Bartelmez and Dekaban, 1962; Hitchcock and Hickey, 1980). The central portion of voluminous thalamic fibers within the cerebral stalk (“Hemispherenstiel” of Hochstetter and His), found in both histological and MR tractographic images, was described in our previous studies. In those studies, we used AChE histochemistry to label TH-C fibers in the frontal (Kostovic and Goldman Rakic, 1983; Kostovic and Rakic, 1984), temporal (Krmpotic-Nemanic et al., 1983), and visual pre-striatal cortices (Kostovic and Rakic, 1984). The massive compact arrangement of TH-C fibers is a characteristic feature of the growth trajectory at the telencephalic-diencephalic borders and more lateral pallial-subpallial border, below the ganglionic eminence. These strategic points along the growth trajectory are essential for initial pathfinding. Moreover, they are considered to be the crucial points of the action for basic axonal-guidance cues (Chen et al., 2012; Jabaudon and López Bendito, 2012; Molnár et al., 2012; Price et al., 2012; Garel and López-Bendito, 2014) and their interaction with cortical efferents to the thalamus (Molnár et al., 1998a,b; Bystron et al., 2006; Grant et al., 2012). The telencephalic-diencephalic border contains a transient fetal structure, the ganglio-thalamic body (Rakic and Sidman, 1969; Letinic and Kostovic, 1997), but its relationship with the outgrowth of the TH-C axons remains unknown. The pallial-subpallial border is important for cortical GABAergic neuron migration (Petanjek et al., 2009), although the interaction of thalamic fibers with GABAergic migratory neurons has not yet been documented at this location. When TH-C fibers pass this crucial morphogenetic border during the early fetal period, they enter another important crossroad area in the periventricular space (Judas et al., 2005). Afterward, they fan out to form the prominent radiation that grows rather directly to the midlateral cortex, showing short distance trajectory in the deep stratum of the intermediate zone. This radiating growth of somatosensory TH-C fibers allows earlier interactions (1–2 weeks) with the transient SP zone and earlier penetration of the CP (around 23 PCW) (Molliver et al., 1973; Kostovic and Rakic, 1984, 1990).

### Developmental interactions with other pathways (callosal fibers, non-thalamic SP, and CP pathways)

One of the most interesting findings in our study is the close interdigitation of radiating TH-C fibers and callosal axon bundles. This spatial relationship between the callosal bundles and TH-C fibers forming the corona radiata is less obvious in the adult brain due to the complexity of fiber arrangement in the centrum semiovale. However, in the fetal brain, where the associative fibers are not yet fully developed and fibers form more discrete bundles, the interdigitation of the TH-C and callosal fibers is prominent in both histological preparations and MR images.

We hypothesized that this prominent barrier of earlier growing TH-C fibers that interdigitate with callosal fibers may explain the paucity of callosal input to some parts of the somatosensory cortex (Killackey and Chalupa, 1986; Jones et al., 1994). In support of this hypothesis, we show histological evidence that during callosal growth, the TH-C fibers use chondroitin-sulfate proteoglycan as a substrate of growth in the SP zone and a substrate for the ingrowth in CP (Bicknese et al., 1994; Kostovic et al., 2002, 2014). Since the chondroitin-sulfate proteoglycans are inhibitory factors for the growth of other non-thalamic fiber systems (Kostovic et al., 2014), their increased concentration in the extracellular matrix (around growing TH-C fibers) may inhibit callosal fibers from approach their target somatosensory cortical area. The TH-C fibers to the somatosensory cortex approach the SP 1–2 weeks earlier than other cortical areas, and this period may correspond to the developmental window necessary for ingrowth of callosal fibers to the somatosensory cortex.

The overlap in the timing of the growth of different fiber systems in the human cerebrum is significant (Rakic, 1999; Kostovic and Judas, 2007; Vasung et al., 2010) and distinguish the development of fiber systems in the large primate brain from those in the rodent brain (Lund and Mustari, 1977; Hoerder-Suabedissen and Molnár, 2015). The prolonged overlap in the period of sequential and overlapping growth opens the developmental possibility of various morphogenetic interactions and complex interactions of transcriptional factors for regulating guidance cues, chemoattractants, and chemorepellents. These complex molecular and chemogenetic interactions between TH-C afferents and the cortex are relatively well-identified in experimental models (Sestan et al., 2001; Chen et al., 2012; Jabaudon and López Bendito, 2012; Molnár et al., 2012; Price et al., 2012; Garel and López-Bendito, 2014). However, developmental interactions of the TH-C system with other fiber systems such as callosal, basal forebrain, and associative fibers remain to be clarified. For example, it would be interesting to determine whether afferents from the cholinergic nucleus basalis (Maynert complex) interact with TH-C axons during concomitant growth and synaptogenesis within the SP zone (Kostovic, 1986; Hanganu et al., 2009). We previously found that TH-C fibers and basal forebrain fibers from the external capsule concomitantly participate in SP expansion (Duque et al., 2016), “wait” jointly in the SP (Kostovic and Goldman Rakic, 1983; Kostovic and Rakic, 1984; Kostovic, 1986), and penetrate the CP after 24 PCW (Kostovic, 1990). Synaptogenesis and functional activity within the transient SP zone also involves both systems: thalamocortical (Molliver et al., 1973; Kostovic and Rakic, 1984, 1990; Shatz, 1992), presumably via glutamatergic activity (Ghosh et al., 1990; Khazipov and Luhmann, 2006; Hanganu et al., 2009; Kanold and Luhmann, 2010), and basal forebrain (Kostovic, 1986), presumably via cholinergic activity (Hanganu et al., 2001, 2009).

Early involvement of thalamic afferents in synaptic oscillatory activity of the SP (Kanold and Luhmann, 2010) opens the possibility that thalamic input plays a role and influences cortical circuitry formation even before direct TH-C synaptic engagement in the CP layers (Molliver et al., 1973; Higashi et al., 2002; Molnár et al., 2012). Thus, functional interaction of thalamic axons in the SP zone may have multiple roles: activity of thalamic axons may promote cortical target selection (Catalano and Shatz, 1998), and thalamic interaction with the SP may influence cortical circuitry formation as documented in the studies on Carnivora (Allendoerfer et al., 1994; Kanold, 2004) and rodents (Hanganu et al., 2002; Dupont et al., 2006; Khazipov and Luhmann, 2006; Pinon et al., 2009; Zhao et al., 2009).

We previously proposed that prolonged existence of the transient SP and permanent cortical circuitry in the CP, which involves the TH-C system of the developing human fetal and preterm cortex (Kostovic and Judas, 2006; Kostovic and Jovanov-Milosevic, 2006), is a salient feature of human cortex development. This phenomenon is probably related to the prolonged differentiation of complex cortical connectivity, especially the late, postnatal differentiation of associated cortical areas (Kostovic and Jovanov-Milosevic, 2006; Kostovic and Judas, 2007; Kostovic et al., 2014). However, recent studies in the developing mouse cortex have demonstrated relatively prolonged functional differentiation of thalamus-SP-layer 4 circuitry and contributions in the development of neocortical organization (Zhao et al., 2009).

The early presence of the anatomical and functional substrates of sensory TH-C circuitry in the fetal and preterm cerebrum is in accordance with both classical (Hrbek et al., 1973; Graziani et al., 1974; Novak et al., 1989) and current studies (Vanhatalo and van Nieuwenhuizen, 2000; de Graaf-Peters and Hadders-Algra, 2006; Vanhatalo and Kaila, 2006; Milh et al., 2007; Nevalainen et al., 2014) showing early development of evoked cortical responses upon sensory stimulation from the environment. From physiological, clinical, and ethical points of view, it is important to note that pain stimuli also evoke cortical responses as early as 26 weeks of gestation (Anand and Hickey, 1987; Anand et al., 1989; Fitzgerald, 1991, 2005; Lee et al., 2005; Slater et al., 2006; Norman et al., 2008; Fabrizi et al., 2011). While it is indeed the case, it is not clear that these stimuli elicit pain sensation at these early stages.

In this respect, it is essential to know whether early TH-C sensory input in preterm infants affects synapse number. The question of early thalamic input from the sensory periphery in prematurely born infants was a constant focus of human developmental neurologists and raises the question of possible influences of the extrauterine environment on cortical circuitry development. Due to the fragile nature of preterm human neonates, the requirements of intensive care, and ethical issues, this problem cannot be studied using a direct approach. That preterm birth is not a normal event and the difficulty defining what is normal in the developing preterm infant contributes to the complexity of the problems. Therefore, we must take into the account the experimental background when determining how sensory thalamic input influences cortical development.

Extensive experimental findings from the rodent somatosensory cortex (Khazipov and Luhmann, 2006; Pinon et al., 2009; Tolner et al., 2012) support the importance of early thalamic input for cortical circuitry formation. In this respect, it is essential to know whether induced prematurity conditions in experimental primates will change the number of synapses. Bourgeois et al. (1983) exposed prematurely born monkeys to precocious visual stimulation but did not observe an increase in synapse number. This important finding suggests that synaptogenesis is an endogenously programmed control that occurs before birth.

### Normative data on thalamocortical development and vulnerability

Precise timing of TH-C growth is essential for studying vulnerability of the TH-C system during the critical developmental “window.” Our results demonstrate that using combinations of histological and MR techniques allows identification of all major growth phases of TH-C somatosensory fibers. Most of our findings on TH-C fiber development during the late fetus/preterm period are in accordance with recent MR studies of the TH-C system performed *in vivo* (Barkovich, 2006; Kasprian et al., 2008; Ball et al., 2012, 2015; Mitter et al., 2015) and *in vitro* (Kostovic et al., 2002; Huang et al., 2006, 2009; Dubois et al., 2008; Takahashi et al., 2012; Wang et al., 2015).

Hypoxic-ischemic lesion of growing white matter is a hallmark of pathology during human fetal and preterm brain development (Volpe, 1996; Krägeloh-Mann et al., 1999; Sie et al., 2000; Hoon et al., 2002; Miller et al., 2002; Counsell et al., 2003; Ment et al., 2009; Miller and Ferriero, 2009; Ball et al., 2013; Bregant et al., 2013; Kidokoro et al., 2014; Kostovic et al., 2014). We recently proposed (Kostovic et al., 2014) that vulnerability of different classes of growing axons in the developing cerebrum mainly depends on two factors: the developmental phase of the growth (pathfinding, “waiting” period, target invasion) and the radial position within the cerebral compartments (deep periventricular, intermediate, and superficial). Regarding the first factor of the developmental growth phase, it seems that TH-C fibers may be vulnerable during growth in the early fetal, midfetal, and late fetal-preterm periods. Data on vulnerability during the early fetal (pathfinding) and midfetal (“waiting”) period in humans are not available. At the end of the midfetal period (between 22 and 24 PCW) during accumulation below the CP and initial penetration of the CP, the vulnerability of the TH-C fibers is topographically related to their position in the periventricular crossroad area (Judas et al., 2005), sagittal strata (Rados et al., 2006; Kostovic et al., 2014), and more distal segments of the sagittal strata (Kostovic et al., 2014). It is important to mention that this is the critical age limit for the survival of prematurely born infants. Furthermore, there are several indicators that this especially vulnerable period could also represent a period of TH-C afferent accumulation within the superficial SP (around 22 PCW). This period is also characterized by an increased need for TH-C fiber growth promoting substrates, such as chondroitin sulfate (Bicknese et al., 1994). This is followed by removal of this substrate in subsequent weeks, which facilitates complex interactions between ECM molecules, axonal receptors, and axonal cues released from the CP and SP neurons. These growth molecules may be vulnerable to factors that develop during hypoxia–ischemia. In addition, hypoxia-ischemia can cause abnormal glial reactivity (Pogledic et al., 2014). However, there are very few reports describing cellular pathology observed in the SP zone (Kinney et al., 2012). Direct evidence for pathological changes of different cellular elements (neurons, glia, ECM, and axons) of the SP during hypoxic-ischemic episodes in the developing human brain is still lacking. Even less is known about hypoxic-ischemic lesions of the “waiting” and accumulating TH-C axons in the SP during this (22 PCW) and subsequent preterm period (23–28 PCW).

Some researchers have proposed that the hypoxia-ischemia damages SP neurons, causing later cognitive impairment due to abnormal development of the cortical circuitry (Volpe, 1996). This type of pathology is particularly interesting in so-called diffuse leukomalacia (Volpe, 1996, 2009). The MR substrate of diffuse leukomalacia is not precisely defined due to the lack of the systematic postmortem studies of prematurely born infants who underwent MR scanning during the course of their intensive care unit (ICU) treatment. One of the most intriguing findings is the observation of white spread changes in MR signal intensity in the developing “white” matter, so-called DEHSI (diffuse excessive high signal intensity; Maalouf et al., 1999; Counsell et al., 2006). These changes were present in about 75% of preterm infants (Maalouf et al., 1999; Counsell et al., 2006). These MR findings led to the attractive hypothesis that this signal abnormality, when prominent, marks a prospective lesion of developing fetal white matter (Counsell et al., 2003, 2006). However, some studies did not find a correlation between neurodevelopmental outcomes and DEHSI in these children (Kidokoro et al., 2011). Specific TH-C system changes in preterm infants were described more recently (Ball et al., 2012, 2013, 2015). In a later period (26–28 PCW) when the TH-C fibers have already penetrated the CP, TH-C fiber lesions may coincide with signal intensity changes in the posterior limb of the internal capsule (Rutherford et al., 1998). This finding is consistent with anatomical data showing that TH-C fibers, upon exiting from the internal capsule, run through the periventricular crossroads and sagittal strata (von Monakow, 1905; Judas et al., 2005; Kostovic et al., 2014). The normal appearance of the crossroad area (Kidokoro et al., 2011) and sagittal strata (Kostovic et al., 2014) on MR scans at the term-equivalent age are good predictors of normal outcome in preterm infants who suffer ischemia. Knowing that TH-C fibers are the prominent component of the sagittal strata in the occipital lobe, one can predict that TH-C lesions will cause changes in the sagittal strata and associated crossroad, both being components of segment II of the white matter (von Monakow, 1905; Judas et al., 2005; Kostovic et al., 2014). Therefore, lesioning of the voluminous TH-C fibers may partly explain the reduction of cerebral volume in infants born prematurely (Counsell et al., 2003; Barkovich, 2006; Ball et al., 2012; Kidokoro et al., 2014).

Based on the existing evidence, we propose that the period of developmental vulnerability of the TH-C fibers exists between 22 and 28 PCW, which is earlier than the vulnerability of the associative fiber system (Kostovic et al., 2014). According to the timing presented here, the developmental window of vulnerability of the somatosensory component of the TH-C system is expected to be 1–2 weeks earlier than that of fibers directed to the frontal and occipital associative cortices. Our results on the phases of TH-C growth underscore the importance of collecting normative data on the development of the transient fetal zones and white matter segments to understand human fetal brain vulnerability (Judas et al., 2005; Takahashi et al., 2012; Kostovic et al., 2014). Considering the early maturation of sensory functions in preterm infants (Hrbek et al., 1973; Fitzgerald, 1991, 2005; Lee et al., 2005; Slater et al., 2006; Vanhatalo and Kaila, 2006; Norman et al., 2008), one can expect serious consequences if TH-C fibers are lesioned during cortical circuitry development (Ball et al., 2012, 2013, 2015). Cortical circuitry reorganization after lesioning of TH-C system may contribute substantially to the complex picture of neurodevelopmental outcomes. Due to the importance of the TH-C system for the development of consciousness (Kostovic and Judas, 2010; Lagercrantz and Changeux, 2010), cognitive development, and general cortical activity (Toulmin et al., 2015), developmental lesions in late fetuses and prematurely born infants may be an important component of the pathogenetic mechanisms underlying neurological, mental, and cognitive disorders including schizophrenia (Anticevic et al., 2014) and autism (Nair et al., 2013). Considering the prospective significance of prenatal lesioning of thalamic-SP connections, it is important to note that the SP transcriptoma contains autism and schizophrenia susceptibility genes (Wang et al., 2010, 2011; Hoerder-Suabedissen et al., 2013). In addition, impairment of thalamic input to the SP may disturb the development of SP neurons and affect their “normal” position and gray-white matter boundaries (Kostovic et al., 2014). Gray-white matter boundary integrity seems to be significantly reduced in autism spectrum disorder (Andrews et al., 2017). In light of these new findings on the developmental origins of autism and schizophrenia, our results help delineate the developmental window of vulnerability of thalamic-SP connectivity during the transition between the second and third trimesters of gestation.

## Ethics statement

Ethics Committee approval of the study was sent previously via email. It was signed by Professor Bozo Kruslin, MD, Ph.D. School of Medicine University of Zagreb, Ethic Committee vice president.

## Author contributions

IK and ŽK designed research, performed research, analyzed data and wrote the paper, LV and HH performed research and analyzed data, VM analyzed data.

## Funding

This work was supported by grants from the Croatian Science Foundation Award (Hrvatska zaklada za znanost, HRZZ IP2014-09-4517) (IK) and Adris Foundation (ŽK). LV was supported by SNSF grant No. P300PB_167804.

### Conflict of interest statement

The authors declare that the research was conducted in the absence of any commercial or financial relationships that could be construed as a potential conflict of interest. The handling Editor declared a shared affiliation, though no other collaboration, with one of the authors LV, and the handling Editor states that the process met the standards of a fair and objective review.
